# Macular function in patients with medium myopia

**DOI:** 10.1007/s10633-022-09907-6

**Published:** 2022-12-04

**Authors:** Ewa Małgorzata Grudzińska, Wojciech Lubiński, Monika Modrzejewska

**Affiliations:** 2nd Department of Ophthalmology, Pomeranian Medical University, ul. Powstańców Wielkopolskich 72, 70-111 Szczecin, Poland

**Keywords:** Myopia, PERG, OCT, AL

## Abstract

**Purpose:**

This work aims at assessing whether electrophysiological functional changes in the macular region appear in medium myopia, even in the presence of a normal macular OCT scan and how axial length correlates with macular OCT parameters in medium myopia.

**Methods:**

The study included right eyes of 17 patients with myopia of medium degree (SE <  − 6D to >  − 3D). Control group consisted of 20 eyes of patients of age and sex that matched healthy controls with normal macular and optic nerve OCT results and normal axial length. Full ophthalmic examination (the distance best-corrected visual acuity, intraocular pressure, refractive error, the anterior and posterior segment of the eye in a slit lamp, the axial length of the eyeball) with OCT of the macular and optic disk and the PERG test were performed in the study and control groups. Only the patients with normal ophthalmic and OCT examination results were qualified. The interview covering questions on risk factors of myopia onset and progression such as prematurity, family history of myopia was carried out in both groups. In myopic group, the question relating to time of near work was also asked. Study and control groups were tested with the use of Shapiro–Wilk, Mann–Whitney, Student’s t test, Pearson and Spearman's rank correlation tests.

**Results:**

AL was significantly longer in myopia group (*p* < 0.01), and SE value was lower (*p* < 0.01). Longer implicit time of P50 was found in the study group, but amplitudes of P50 and N95 waves were not significantly reduced (*p* < 0.05). AL showed correlations with P50 implicit time (*p* < 0.05) and with reduction in retinal fiber nerve layer and ganglion cells and inner plexus layer (*p* < 0.05).

**Conclusion:**

Patients with myopia of medium degree have a dysfunction of retinal cone system of the macular region even when OCT scans show no abnormalities. Elongation of AL correlates with reduction in retinal fiber nerve layer and ganglion cells and inner plexus layer. Longitudinal follow-up studies may answer the question whether this increase in implicit time may be indicative of a faster myopia progression or of myopic retinal pathology, i.e., whether it may help to determine which patient would benefit from earlier or more intensive management of myopia progression*.*

## Introduction

Myopia is a serious social problem due to significant increase in the incidence thereof, particularly in Asian countries, but also in Poland. The risk factors of myopia development and progression include myopia in parents, near work, high level of education, age of myopia onset or improper diet [1–3]. There are results published which show that the consumption of refined carbohydrates significantly increased the probability of myopia occurring in girls, but decreased in case of boys [4]. A high degree of myopia is associated with the risk of vision-threatening complications such as myopic macular degeneration, retinal detachment, cataract or glaucoma, but can also occur in a lower degree of myopia [2]. The current state of knowledge does not allow to determine unambiguously the early signs of such changes in people with normal fundus appearance.

Electrophysiological retinal studies are of great value in clinical practice and research due to the possibility of detecting of early functional changes that appear before the occurrence of structural changes [5]. One of the most common complications in myopia is myopic macular degeneration. The risk of the occurrence thereof raises with the increase in value of refractive error [6]. It is important to detect patients with a high risk of irreversible macular changes. We could probably recommend methods of slowing down the progression of myopia to these patients and, therefore, decrease the risk of this complication. Pattern electroretinography (PERG) is the examination which could help with the selection of patients endangered with macular degeneration because it provides information on functioning of ganglion cells, as well as cone photoreceptors, bipolar cells within 15 central degrees of the retina [7].

PERG was chosen, because it shows macular function of ganglion cells and external layers of retina. It also gave us the possibility to distinguish between macular dysfunction and neuropathy.

In the literature, a few electrophysiological studies using PERG were performed in myopia [8–11]. However, within these studies, patients with and without myopic maculopathy were not analyzed separately. These studies suggested disturbances of retinal bioelectrical function. However, there is only the single report indicating the occurrence of macular dysfunction assessed by the PERG in patients with medium degree myopia [12] without visible fundus changes (no OCT testing), which suggest its prognostic value. This study was performed on a small number of cases and was not correlated with the structural changes. Up to date, no study resulted in correlating structural changes with macular function. That's why, we decided to perform PERG in medium myopia. We particularly wanted to focus on patients without degenerative changes in macular OCT. Therefore, it is interesting whether the assessment of macular cell function can be a predictive factor of myopia progression, especially in eyes which do not show structural changes detected with the optical coherence tomography (OCT). This work aims at assessing whether electrophysiological functional changes in the macular region appear in medium myopia even when macular OCT scan is normal and how axial length correlates with macular OCT parameters in medium myopia.

## Material and methods

The study included right eyes of 17 patients (12 females, 5 males) aged 29.1 ± 4.64 years with myopia of medium degree diagnosed in the outpatient clinic of 2nd Department of Ophthalmology. The spherical equivalent of the refractive error was  − 4.48 ± 0.95D on average (range  − 3D– − 6D). The length of the eyeball in the examined group was elongated and the mean value was 25.05 mm ± 0.74. The exclusion criteria were refraction error expressed as SE <  − 6D or >  − 3D, presence of concomitant diseases both ophthalmologic and systemic with known influence on retinal function, taking any medications, history of surgery, strabismus, amblyopia, lesions on the eye fundus except for a mosaic pattern of the retina and slight peripapillary atrophy, as well as structural disturbances in OCT examination. The age- and sex-matched control group consisted of healthy individuals aged 20–40 years with refractive error ± 1D, with mean AL23.27 ± 0.78 and SE 0.03 ± 0.52D. The control group consisted of patients without history of prematurity and myopia in parents. In the study and the control groups, the following tests were performed: interview (including risk factors of myopia occurrence and progression such as prematurity, family history of myopia), the distance best-corrected visual acuity (DBCVA, logMAR), intraocular pressure (Pascal, Swiss), refractive error (Autorefractometer Topcon KR-800, Tokyo, Japan) after cycloplegia with 1% Cyclopentolate, the anterior and posterior segment of the eye in a slit lamp (Volk 90D), the axial length of the eyeball (IOL Master 700, Carl Zeiss, Meditec AG, Jena, Germany), the structure and thickness of the macula and optic disk (Zeiss CIRRUS 500 HD-OCT Carl Zeiss Meditec AG, Jena, Germany) and the PERG test (RetiPort Roland Consult GmbH, Germany). In myopic group, we also asked questions about time of near work.

PERG test was performed in accordance with the ISCEV standards [13].

The test was performed in standard room lighting. A 21'' cathode ray tube monitor with a refresh rate of 75/s and screen lumination of 120 cd/m^2^ was used. The reversing black and white checkboard pattern was a stimulus. The size of a single square was set to 0.96°, and the image contrast was 97% (modulation-reversal mode). The stimulation frequency was 2.35 Hz, i.e., 4.7 changes/sec. The test was performed in a sitting position after correcting the refractive error for a test distance of 0.5 m. The pupils were not dilated for the test. The examination was carried out unilaterally with central monitor fixation. The active electrode (DTL) was placed along the edge of the lower eyelid, passive electrode, gold cup electrode, placed near the outer canthus of the eye and ground electrode, gold cup electrode, was placed in the middle of the forehead. The impedance of electrodes was < 5kΩ. In each eye, two series of records of 200 runs were made, which were then averaged and then averaged off-line. The recording frequency range was 1–100 Hz and the analysis time 250 ms with the notch filters switched off. The amplitudes of P50 and N95 waves and the P50 implicit time were evaluated. The P50 amplitude was measured from the trough of N35 to the peak of P50. The N95 amplitude was measured from the peak of P50 to the trough of N95.

The study received approval of the Bioethics Committee at the Pomeranian Medical University (Consent No. KB-0012/154/17). The study protocol was in compliance with the requirements of the Helsinki Declaration. Prior to the study, written consent to the study was received from each participant. The patients were informed about possible complications related to the examination.

The results of the PERG examination in the study group and the control group were analyzed statistically. Moreover, correlations between the results of the PERG examination in the study group and risk factors of myopia progression were analyzed. The Shapiro–Wilk test was used to assess normal distribution. Tests were used to compare groups: Mann–Whitney’s for variables without normal distribution and the Student’s t test for normal distribution. The Pearson and Spearman’s rank correlation analysis (for normal distribution and non normal distributions, respectively) was carried out to assess the relationship. The value of P < 0.05 was considered statistically significant. P values for both, comparisons and correlations were corrected using Holm’s sequential Bonferroni procedure.

## Results

According to the inclusion criteria, patients with normal ophthalmic and OCT examination results were qualified. Interview data are presented in Table 1.

**Table 1:** Table showing the occurrence of risk factors of myopia onset and progression in the study group

Risk factor	Myopic group	Control group
Age of myopia onset	Average 12.34 years ± 3.0	–
Time of near work (hour/day)	Median 8 h/day (2–12 h/day)	No data
Prematurity	6 (18.7%)	0
Time of physical activity (hours/week)	2 h/week (0–5 h/week)	3 h/week (0-10 h/week)
Myopia in one parent	14 (43.8%)	0
Myopia in both parents	6 (18.7%)	0

No significant differences between age, DBCVA or IOP of groups were found (Table 2). AL was significantly longer in myopia group (*p* < 0.01), and SE value was significantly lower (*p* < 0.01). The results of the PERG and OCT are presented in Table 2.

**Table 2:** General characteristics of groups and results of PERG and OCT test

	Study group SE (− 3D do − 6D) *n* = 17	Control group SE ± 1D *N* = 20	p
Age	29,1 ± 4.64	28.9 ± 3.3	0.86**
DBCVA	M -0.14 (-1 – 0.02)	M − 0.16 (− 0.28- 0.18)	0.48*
SE [D]	-4.48 ± 0.95	0.03 ± 0.52	0.00000**
AL [mm]	25.05 ± 0.74	23.27 ± 0.78	0.00000**
IOP [mmHg]	16.29 ± 1.5	15.42 ± 2.5	0.24*
P50 implicit time	52.20 ± 2.0	M 49.78 (46.20–53.35)	0.007*
P50 amplitude	4.73 ± 2.04	M 5.25 (3.1–8.8)	0.24*
N95 amplitude	6.89 ± 2.59	7.89 ± 2.4	0.25**
RNFL [µm]	89.40 ± 5.78	97.60 ± 5.37	0.0001**
GCIPL [µm]	79.87 ± 3.74	85.30 ± 3.45	0.00009**
CST [µm]	266.60 ± 16.35	260.10 ± 21.74	0.34**

Comparison of a study group with a control group—Mann–Whitney test(*) or Student t test (**). Results mean ± standard deviation, M-median, in parenthesis minimum and maximum range. Bold statistically significant values. *DBCVA* best-corrected distance visual acuity, *SE* spherical equivalent of refractive error, *AL* axial length, *IOP* intraocular pressure, *RNFL* retinal nerve fiber layer, *GCIPL* ganglion cell and internal plexus layer, *CST* macular central subfield thickness

**Fig. 1:**
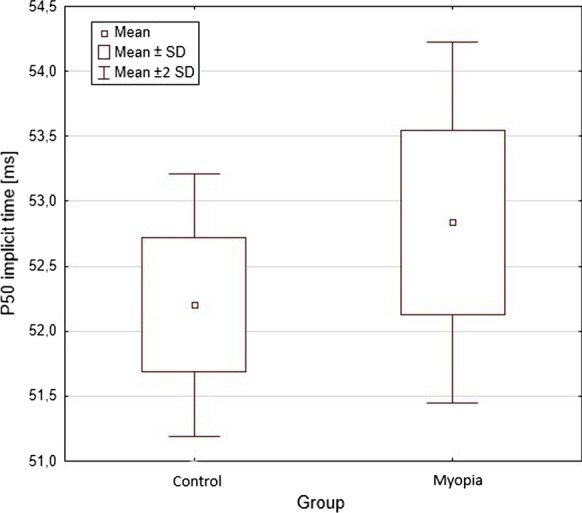
Scatterplot of P50 implicit time by groups

Significantly longer implicit time of P50 wave was found in the group of eyes with myopia—Fig. 1. Amplitudes of P50 and N95 waves were reduced in the group of eyes with myopia, but the differences did not reach statistical significance (*p* > 0.05).

We found several correlations between structural changes in OCT and axial length/degree of myopia and PERG (Table 3).

**Table 3:** Correlations between structural changes in OCT and axial length/ spherical equivalent and PERG

Correlations	SE	AL	P50 amplitude	N95 amplitude	P50 implicit time
RNFL	**0,63**	− **0,52**	**0,31**	**0,29**	− 0,16
GCIPL	**0,71**	− **0,65**	**0,50**	**0,49**	− **0,30**
CST	− 0,14	**0,29**	− **0,28**	− **0,29**	0,16

R values in table. Bold statistically significant values. *SE* spherical equivalent of refractive error, *AL* axial length, *IOP* intraocular pressure, *RNFL* retinal nerve fiber layer, *GCIPL* ganglion cell and internal plexus layer, *CST* macular central subfield thickness

**Fig. 2:**
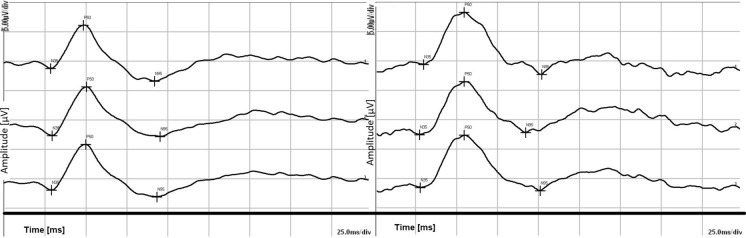
Example of PERG result in the control group (left) and study group (right)

When PERG examination of the myopic patient was analyzed and compared to normal controls, six eyes with abnormal results were recorded in the study group (35%, 6/17 eyes). The most frequent features detected were the discrepancies relating to implicit time of P50 wave (6 eyes), reduction in the amplitude of P50 wave (1 eye), reduction in the amplitude of N95 wave (1 eye) and reduction in the amplitude of P50 and N95 wave (1 eye). Figure 2 presents the PERG result of the patient with myopia (right) with a significant increase in the P50 implicit time in comparison with normal control eye of a patient (left).

**Fig. 3:**
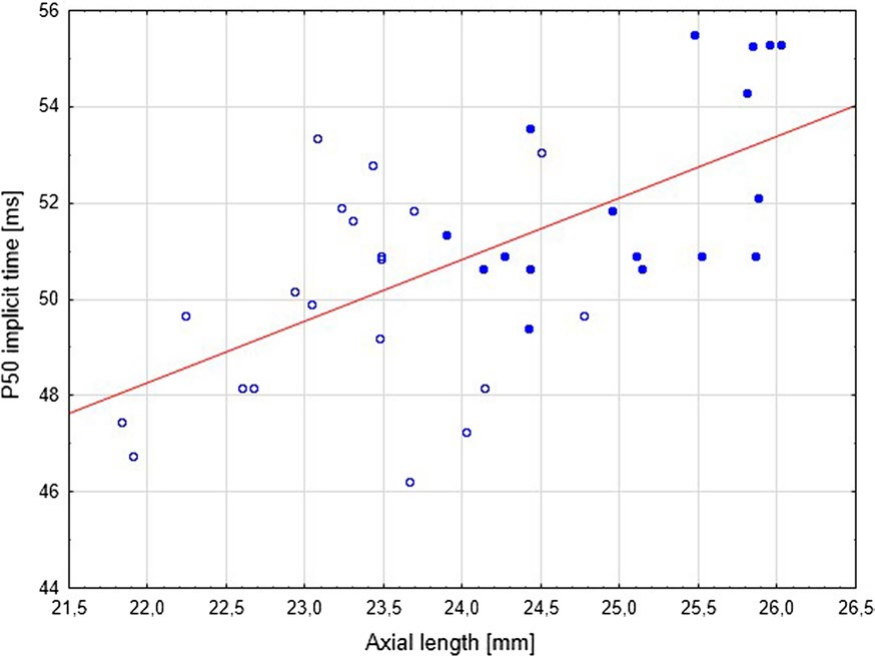
Correlation of AL and implicit time of P50—Spearman's ranks (*p* < 0.05 *r* = 0568). Legend: full dots—myopic patients, white dots—control group

SE and AL showed correlation with P50 implicit time (*p* < 0.05 *r* =  − 0.51). No statistically significant correlation was found between the age of the onset of myopia, the age of myopia stabilization and the amplitude and implicit time of P50's or the N95's amplitude. There was also no significant correlation between the prematurity and PERG results and between myopia in parents and the PERG results (*p* > 0.05). None of the patients underwent laser or cryo treatment (Fig. 3).

## Discussion

In the present study, for the first time, it has been shown that the abnormal bioelectric function of the central retina measured by PERG (transient type) in subjects with medium axial myopia may occur despite the lack of structural changes in the macular and optic nerve disk region in the OCT. The only statistically significant change was the prolongation of P50 implicit time—Table 2. There is a study available, the results of which indicate that patients with myopia of medium degree may have retinal ganglion cell dysfunction recorded in PERG (steady-state type). A reduction in peak-to-peak PERG values was found in 30% of patients with myopia of medium degree [12]. And the frequency of abnormal results was similar to our results (28%). However, in the study mentioned above, the structural changes in the posterior pole of the eye were not assessed. Therefore, it could not be excluded that the obtained alterations in PERG were a consequence of not only functional but also structural changes in the ganglion cells. Similar problem with lack of information about structural changes relates also to another study that showed prolongation of P50 implicit time in patients with medium myopia without significant changes of P50 and N95 amplitudes [11].

In the present study, changes of ganglion cell function could not be excluded, because in addition to a significant increase in the implicit time of the P50 wave, the trends (at the borderline of statistical significance) of reduction in the amplitudes of P50 and N95 waves are observed—Table 2. *The OCT normal values were defined by the device software. Image showed no structural damage, and all the layers were present and not interrupted, but statistical analysis showed the GCIPL and RNFL layers were thinned in myopes, when we compared myopic group with normal group.* Based on these results, we suggest that reduction in P50 and N95 amplitudes could be caused by thinning of RNFL and GCIPL in our study group. A larger study group would confirm this suggestion. From the available literature, it is known that the increase in the P50 wave implicit time is not characteristic for ganglion cell damage, but is rather associated with the dysfunction of the external retina in relation to the ganglion cells (cone photoreceptors) which occur in macular diseases [14]. We hypothesize that the first detectable change is the dysfunction of cone photoreceptors of the macular region.

Another evidence indicating the possibility of cone system dysfunction are the results of Chen et al. who, in myopia up to -10D, found an elongation of P1 wave culmination time in mfERG [5]. It is known that the prolongation of P1 wave culmination time is associated with damage to the function of cone photoreceptors [7].

Several theories were proposed to explain the etiology of myopia progression [15, 16]. Therefore, we focused on theory of reduced level of dopamine. Changes in the dopaminergic system make up one of the possible hypotheses explaining the dysfunctions of the cone system in myopia of medium degree. Changes in the dopaminergic system occurring in myopia have been described in the literature [17]. Dopamine levels are believed to increase the length of the eyeball [16]. In studies with animals, it has been observed that chickens with experimental myopia have decreased dopamine levels [18]. Another study on mice, showing the role of dopamine in myopia development, highlights the role of bright light in stopping myopia progression based on increased dopamine receptor activity [19].

This relationship was also noted in human studies where light stimulation increased dopamine secretion and inhibited myopia development [16, 20]. Another evidence of the important role of increased dopamine levels in inhibiting myopia is spending time outdoors. It has been calculated that the risk of myopia decreases by 2% per additional hour of the week spent outdoors [21].

Dopamine receptors can be found in the cone receptors [22]. The highest concentration of cones is in the posterior pole of the eyeball and PERG examination shows function of this region. PERG is the examination which shows not only the function of ganglion cells, but as well cones.

In clinical trials, neuropsychiatric diseases are the additional evidence that low levels of dopamine can affect dysfunction of the cone system. As during the embryogenesis, the retina is formed from the same germ leaf as the brain, and these tissues share many common features including the type of neurotransmitters and receptor type [23]. In major depression, neurotransmission changes depending on dopamine, serotonin and norepinephrine are described [24], which can be registered with the PERG examination [24, 25]. Another disease involving the loss of dopaminergic neurons is the Parkinson's disease or Alzheimer’s disease [26–30]. The above-described changes in the PERG examination, manifested by a decrease in the amplitude of P50 and N95 waves as well as an increase in the P50 implicit time in neuropsychiatric diseases, are related to, among others reduced dopamine levels in the retina [31].

Another evidence stemming from experimental studies performed on quails which lowered the dopamine levels within the retina by using a 6-hydroxydopamine injection manifests itself in prolonged implicit time and decreased maximum response amplitude in the PERG study (steady-state), as well as in myopia progression [31].

The weakness of our study is the impact of refraction on PERG results. Image is made smaller by a myopic correction placed in front of a patient. In the available literature, no influence of glasses myopic refraction correction on the amplitude of transient pattern of ERG waves is known. From the results of other studies, it is known that in patients with axial myopia, as in our study, the concentration of photoreceptors is reduced in posterior pole [5]. It seems sensible to hypostatize that such reduction is responsible for diminished answers of PERG recording. It is evident from the literature that amplitude of P50 and N95 is maximal between 0.75° and 1° stimulus field sizes [32].

The influence of changes in image size according to the refractive lens used was calculated. Lens with the refractive power of -6dpt would reduce a square pattern of 0.96° to 0.89° which appears too small a difference to explain the results observed. This effect needs further research, however, most probably, it has low influence on the findings of this study.

In summary, on the basis of our observations and reports in the literature, our findings indicate an increase in implicit time of the P50 in patient with myopia of medium degree, even when the OCT scan is within normal limits. Longitudinal follow-up studies may answer the question whether this increase in implicit time may be indicative of a faster myopia progression or of myopic retinal pathology or maculopathy to develop and whether this may help to determine which patient would benefit from earlier or more intensive management of myopia progression.

## Data Availability

The data may be obtained from the correspondence author upon reasonable request.
